# The role of aerobic and resistance exercise for cancer cachexia management – A systematic scoping review

**DOI:** 10.1016/j.apjon.2025.100748

**Published:** 2025-06-30

**Authors:** Romane Peyrachon, Astrid Lièvre, Amélie Rébillard

**Affiliations:** a Movement, Sport and Health Laboratory M2S-EA7470, University of Rennes 2, Rennes, France; b Department of Gastroenterology, Rennes University Hospital, Rennes 1 University, INSERM U1242, Rennes, France; c Institut Universitaire de France, Paris, France

**Keywords:** Cancer of digestive system, Exercise, Cachexia, Sarcopenia, Muscle mass

## Abstract

**Objective:**

Cancer cachexia is highly prevalent in digestive and head and neck cancer patients who often face mechanical eating difficulties and metabolic disturbances. However, terminological and diagnostic ambiguities surround cachexia, limiting the evaluation of the effects of management interventions for these patients. Whole-body exercise plays a key role in mitigating weight and muscle mass losses. This scoping review provides an updated synthesis of the effects of exercise interventions and offers practical guidelines for patients with digestive or head and neck cancers experiencing undernutrition, anorexia, sarcopenia, and/or cachexia.

**Methods:**

In accordance with Preferred Reporting Items for Systematic Reviews and Meta-Analyses (PRISMA) recommendations, a systematic search of the published literature was carried out in the PubMed, Google Scholar, and EBSCOhost databases. Studies eligible for inclusion investigated the feasibility and effects of whole-body exercise interventions in digestive or head and neck cancer patients with altered nutritional status. The findings from each study were tabulated and synthesized according to the type of exercise intervention.

**Results:**

Twenty-six full texts out of 603 studies met the inclusion criteria, encompassing a total of 1936 patients participated (mean age: 63.80 ​± ​6.22 years; 822 [31.62%] women). Exercise interventions did not result in adverse outcomes. The most assessed parameters included functional and aerobic capacity, muscle strength, body weight, quality of life, and physical activity levels, with findings ranging from positive effects and trends to non-significant changes. Interventions incorporating endurance exercises demonstrated greater improvements in functional and aerobic capacity compared to those focusing solely on resistance training. Supervised interventions yielded the most significant improvements.

**Conclusions:**

Exercise interventions are safe and feasible for patients with digestive and head and neck cancers identified as suffering from undernutrition, anorexia, sarcopenia, and/or cachexia. Future research should focus on tailoring exercise characteristics to achieve greatest benefits in patients with cachexia. Studies should also explore real-life implementation strategies to optimize patient engagement and adherence while accommodating medical and personal constraints.

**Systematic review registration:**

CRD42024602857.

## Introduction

Cancer cachexia is a multifactorial, irreversible syndrome that progressively develops during both the disease and its treatment.^1^ It leads to a loss of skeletal muscle mass, with or without loss of fat mass, resulting in functional impairments, reduced treatment response and shorter survival.^2^^,^^3^ This condition also negatively impacts quality of life (QoL), social relationships and exacerbates fatigue.^4^ Higher prevalence is observed in digestive (around 80% of patients) and head and neck cancers (around 70% of patients), which directly interfere with food absorption and digestion.^2^ Patients with lung cancer may also experience cachexia, even in the absence of eating difficulties.^5^ Thus, the prevalence of cancer cachexia is high, but it cannot be reversed by conventional nutritional support alone.^6^ Current recommendations call for multimodal management combining pharmacology, nutrition and physical activity/exercise.^7^ Nevertheless, further research is needed to identify relevant solutions and establish more definitive recommendations.^8^

A major challenge to identifying therapies is the difficulty in diagnosing cancer cachexia. Today, diagnosis relies on weight loss criteria, which may be combined with additional markers such as muscle mass and strength, walking assistance, chair rise ability, stair climbing/falls, appetite scores (insofar as a reduction in ingesta may precede weight loss^9^) and blood markers.^8^^,^^10^ However, the lack of consensus complicates the diagnosis of cancer cachexia. Moreover, confusion exists between terms such as anorexia, cachexia, malnutrition and sarcopenia.^11^ Consequently, in the clinical context, this terminological overlap hinders diagnosis and management,^12^ and in the research context, it limits the ability to evaluate the effects of interventions in cachectic patients and the aggregation of their results to establish guidelines.

A handful of publications attempt to establish exercise recommendations for cachexia management. The systematic review by Cheung et al.^13^ identified 12 studies looking at the effects of exercise in cachectic patients. Their analysis showed a trend towards positive effects of exercise on weight, muscle strength and functional performance. However, some of these studies combined cachectic and non-cachectic patients. Given the high prevalence of cancer cachexia in digestive and head and neck cancer patients and the terminology confusion, some studies may have included individuals who were cachectic or at high risk without explicitly labeling them as such. In addition, it is important to differentiate between swallowing exercise interventions—designed specifically for patients with head and neck cancers—and whole-body exercise interventions, which aim to increase overall physical activity and energy expenditure. Swallowing exercises have demonstrated benefits for muscle mass, body weight, oral intake, QoL, and depressive symptoms.^14^^,^^15^ In contrast, whole-body exercises elevate energy demands and may worsen the energy imbalance and weight loss observed in cachectic patients.^16^ Evaluating the impact of whole-body interventions is therefore critical for both head and neck and digestive cancer populations. In clinical practice, such exercise programs are often delivered to patient groups with highly heterogeneous characteristics.

This systematic review aims to provide an up-to-date analysis of the effects of whole-body exercise interventions for patients with digestive and head and neck cancers diagnosed with malnutrition, sarcopenia and/or cachexia. Our findings will help to define exercise management recommendations for these patients.

Given the characteristics of cachectic patients and current evidence, resistance training alone is expected to yield greater benefits in terms of body weight, muscle strength, and muscle mass than combined resistance and endurance training, due to the increased energy demands of the latter. Furthermore, considering patients' fragile energy balance, excessively frequent, prolonged, or intense sessions may compromise the beneficial effects of exercise. Supervised sessions are likely to enhance outcomes by promoting an optimal training load conducive to physiological adaptation. However, their implementation may face lower adherence rates due to logistical constraints.

## Methods

This systematic review was conducted in accordance with the Preferred Reporting Items for Systematic Reviews and Meta-Analyses (PRISMA) guidelines.^17^ This review is registered on PROSPERO (ID: CRD42024602857).

### Information sources

Three databases were searched: PubMed, Google Scholar, and EBSCOhost (which includes MEDLINE, Academic Search Premier, SPORTDiscus with Full Text, Psychology and Behavioral Sciences Collection, and eBook Collection) from their inception until 23/02/2025. Keywords were defined using the PICO method^18^ (Supplementary Material 1). The search strategy included the following MeSH terms and synonyms : “Cancer of digestive system”, “Cancer of head and neck”, “Cachexia”, “Sarcopenia”, “Malnutrition”, “Exercise”, “Rehabilitation”. See Supplementary Material 1 for the detailed search strategy keywords.

No restrictions were applied regarding date or study location. Additional articles were identified manually by reviewing the references of included studies. All articles had to be written in English or French.

The search equations are available in Supplementary Material 1. We verified that no systematic reviews on this topic were currently in progress by consulting the PROSPERO register. As of 23/02/2025, no systematic reviews on the effects of exercise interventions in patients with digestive cancers at risk of cachexia or who are cachectic were found.

### Study selection

Articles from the different databases were uploaded to Rayyan software, and duplicates were removed. Study selection was conducted by two reviewers (RP and AR) through a two-step process. In cases of uncertainty, a third reviewer was consulted to reach a decision. In the first phase, the title and abstract of each article were screened. Studies were considered for the second phase if the title or abstract indicated that the intervention was exercise, rehabilitation, physical activity, or training in cancer patients. The second phase involved a full-text review. Full texts were accessed via Open Access, or through publishers partnered with the laboratory and university. If necessary, full texts were requested from the authors. Inclusion and exclusion criteria for the screening process are presented in the table below (Table 1).

**Table 1:** Inclusion and exclusion criteria for study screening.

Inclusion and exclusion criteria	
Article type	
Inclusion: Clinical trials : Research that compared the exercise intervention with usual care (i.e., randomized control and quasi-randomized control), participants serving as their own control (i.e., longitudinal evaluation of cohort and case studies).	Exclusion: Reviews and opinions
Language	
Inclusion: English, French	Exclusion: Other language
Population	
Inclusion: Malnourished, sarcopenic and cachectic digestive and head and neck cancer patients. Age: Adult patients (≥ 18 years). Diagnostic for cachexia/nutritional alterations could be based on Fearon or EPCRC criteria, weight or muscle mass variations, muscle mass index, questionnaires (PG-SGA, NRS, MNA, GNRI (with variable thresholds [HAS, 2021])).	Exclusion: Animals, healthy population, other chronic diseases, patients without head and neck or digestive cancers, pediatric cancers, cancer patients without nutritional status presented.
Intervention	
Inclusion: Whole-body exercise. Exercise program with or without supervision. Exercise program could contain endurance training and/or resistance training and/or mobility training. Exercise could be delivered through technology devices (videoconference, virtual reality, phone call) or physiotherapist or leaf. Program should length at least 2 weeks.	Exclusion: Acute exercise intervention (less than 2 week), no exercise intervention, swallowing exercise intervention only (which target cancer of head and neck).
Outcomes	
Inclusion: Physiological or psychological outcomes reported. Studies assessing the feasibility of exercise intervention were included if they reported at least one physiological or psychological outcome. Feasibility outcomes included: Eligibility rate, recruitment rate, attrition rate, compliance and adverse events. Physiological outcomes included PA level, motor functions, endurance, strength, mobility, balance, functional capacity, aerobic capacity, body stature (weight, BMI and body composition (LM, FM, MM). Psychological outcomes included nutrition, CRF, QoL, pain, anxiety, and depression, and well-being.	

EPCRC, European Palliative Care Research Collaborative; PG-SGA, Patient-Generated Subjective Global Assessment; NRS, Nutrition Risk Screening; MNA, Mini Nutritional Assessment; GNRI, Geriatric Nutritional Risk Index; HAS, hyperarousal scale; BMI, Body Mass Index; LM, lean mass; FM, fat mass; MM, muscle mass; CRF, cancer-related fatigue; QoL, quality of life.

### Data extraction

A standardized data extraction form was developed in Microsoft Excel specifically for this systematic scoping review. The form was used to record key study information, including citation details (first author, year of publication, study title, and country), study design, and methodological characteristics. Data pertaining to participant demographics were also extracted, such as sample size, mean age, sex distribution, cancer type, treatment status, weight variation, and/or nutritional condition.

Information regarding the exercise intervention was collected in detail, including training modality (aerobic, resistance, or combined), frequency, intensity, duration, session length, timing relative to treatment, and whether sessions were supervised. Where applicable, concomitant interventions such as nutritional support, psychological counseling, or pharmacological treatment were also recorded.

Primary outcomes were extracted at baseline and post-intervention. These included.

1. Safety, assessed by the occurrence of adverse events (AEs) and serious adverse events (SAEs). Reporting and evaluating AEs allowed for the assessment of the intervention's tolerability and potential risks.^19^
2. Feasibility, evaluated through eligibility, recruitment, and completion rates. The eligibility rate was defined as the proportion of patients who met inclusion criteria relative to the total number of patients screened. The recruitment rate referred to the percentage of eligible participants who consented to participate. The completion rate was calculated as the proportion of participants who completed post-intervention assessments.
3. Compliance, defined as the proportion of exercise sessions attended relative to those prescribed.

Additional outcomes of interest included measures of physical (e.g., strength, endurance, body composition) and psychological (e.g., QoL, mood) effects of the intervention, where reported.

### Study quality assessment

Study quality was assessed by two reviewers (RP and AR) using the Physiotherapy Evidence Database (PEDro) scale. This scale is a valid tool for assessing the risk of bias in clinical studies, regardless of design.^20^ It provides a 10-point score based on 11 “Yes-No” questions. The list of questions is available on the PEDro website. A lower score indicates poor quality, and a higher score indicates high quality. PEDro scores of 0–3 are considered ‘poor’, 4–5 ‘fair’, 6–8 ‘good’, and 9–10 ‘excellent’. Furthermore, for trials evaluating complex interventions, such as exercise interventions, a PEDro score of 8/10 is considered optimal.^21^

### Effect measures and data synthesis

The data were summarized in tables and reported in terms of positive, negative, or non-significant effects on the outcomes at the end of the exercise program. Due to substantial clinical, statistical, and methodological heterogeneity between the studies, no meta-analysis could be conducted. *P*-values are reported. A positive effect corresponds to an improvement at the end of the exercise program and/or a significant difference between the intervention and control groups, favoring the intervention group. Intervention effects were examined in relation to key characteristics of the exercise programs, including exercise type, session frequency, intervention duration, intensity, and supervision—collectively referred to as the FITTS framework. In addition, analyses were stratified by patient characteristics, such as tumor type, age (above or below the overall mean age), and proportion of female participants (above or below the sample mean).

## Results

### Study selection

**Fig. 1:**
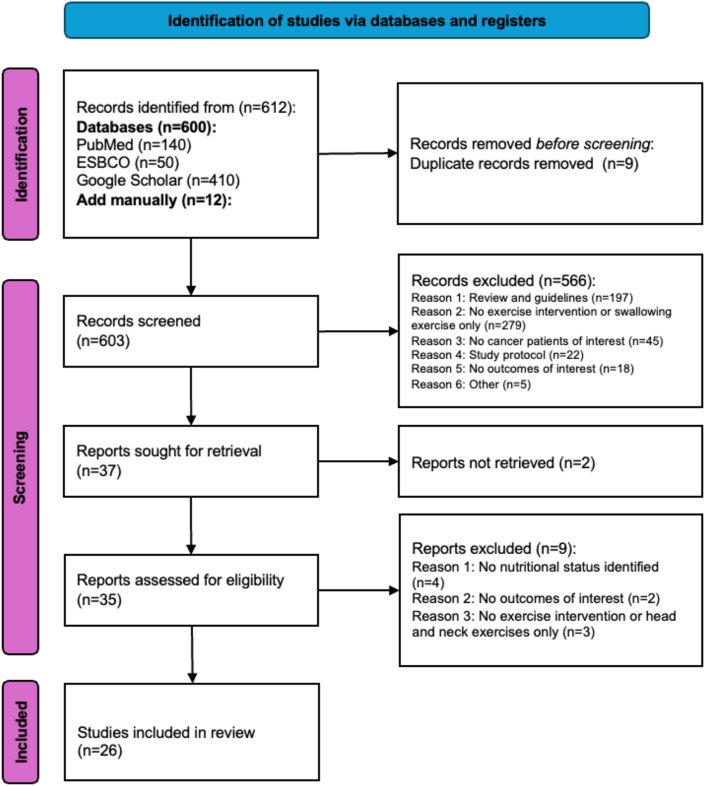
PRISMA flowchart of the article selection process. PRISMA, Preferred Reporting Items for Systematic Reviews and Meta-Analysis.

Our search identified 612 references, from which 9 duplicates were removed. We manually added 12 studies. After screening the titles and abstracts, 566 studies were excluded. The remaining 37 references were deemed potentially relevant, and we proceeded with full-text screening. Two studies could not be retrieved, despite attempts to contact the authors, from whom we received no response. Following the full-text screening, 9 articles were excluded. In total, 26 studies were included in the review (Fig. 1).

### Study characteristics

Study characteristics of randomized controlled trials (RCTs) and Non-RCTs (NRCTs) are summarized in Table 2. A total of 26 studies were included: 14 were RCTs and 12 were NRCTs. The study by Lonbro et al.^22^ was considered an NRCT as both groups underwent resistance training and randomization was to the nutritional intervention. These studies were conducted between 2011 and 2024 in 14 different countries: 5 studies from Germany, 2 from Canada, 3 from Australia, 2 from the United States, 2 from Switzerland, 2 from Japan, 2 from Denmark, 2 from France, 1 from the United Kingdom, 1 from Egypt, 1 from Norway, 1 from Sweden, 1 from Italy, and 1 from Spain.

**Table 2:** Study characteristics.

Study	Population, nutritional status and cancer treatment	Exercise intervention	Exercise characteristics	Additional intervention(s)
Cappozi et al., 2016^24^ (Canada) - RCT	60 head and neck cancer patients Surgery and RT or CT Age: 55.9 ​± ​9.4 (intervention), 56.4 ​± ​9.2 (control) Sex: 5W/26M (intervention), 6W/23M (control) Nutrition: PG-SGA: 6.6 ​± ​5.8 (intervention), 5.9 ​± ​4.9 (control)	Resistance training 12 or 24 weeks depending on the group allocation 4 sessions / week: 2 supervised sessions ​+ ​2 home-based sessions	10 exercises targeting major muscle groups 2 sets of 8–10 reps. Moderate intensity/ Progression at weeks 4, 6 and 9 as appropriate	Health education Behavior change support
Grote et al., 2018^25^ (Germany) - RCT	20 head and neck cancer patients RT only or RT ​+ ​CT or Surgery ​+ ​RT Age: 60.9 ​± ​11.3 (intervention), 61.5 ​± ​15.7 (control) Sex: 1W/9M (intervention), 4W/6M (control) Nutrition: Body weight variation in 6 months: −7.1 ​± ​5.2%	Resistance training During and after radiotherapy 3 supervised sessions of 30 min / week	3 exercises (chest press, leg press and latissimus pull down) 3 sets of 8–12 reps. RPE < 7/10	X
Hall et al., 2021^26^ (UK) - RCT	45 various tumor types: Gastrointestinal (*n* ​= ​18), Thoracic (*n* ​= ​3), breast (*n* ​= ​6), Urological/Gyn (*n* ​= ​10), Myeloma (*n* ​= ​5), head and neck (*n* ​= ​1), endocrine (*n* ​= ​2) End of the treatment Age: > 65 yo (*n* ​= ​14), 55-65 yo (*n* ​= ​3), < 55 yo (*n* ​= ​6) (intervention), > 65 yo (*n* ​= ​16), 55-65 yo (*n* ​= ​4), < 55 yo (*n* ​= ​2) (control) Sex: 9W/14M (intervention), 10W/12M (control) Nutrition: Body weight variation in 6 months: Loss 0–5% (*n* ​= ​10), loss > 5% (*n* ​= ​7), Unknown (*n* ​= ​4) (intervention), loss 0–5% (*n* ​= ​5), loss > 5% (*n* ​= ​9), Unknown (*n* ​= ​6) (control).	Resistance and endurance training 8 weeks 3 home-based resistance training sessions / week + 60 min / week of endurance training (walking)	3 exercises (press ups, half squats and shoulder thrusts) Moderate (RPE 3–4/10)	Nutritional (ONS ​+ ​counselling)
Kamel et al., 2020^27^ (Egypt) - RCT	40 pancreatic cancer patients Surgery and CT Age: 51.6 ​± ​5.1 (intervention), 52.2 ​± ​4.9 (control) Sex: 8W/12M (intervention), 6W/14M (control) Nutrition: Cachexia (Fearon): 100%	Resistance training ​+ ​Mobility 12 weeks 2 supervised sessions of 60min / week	8 exercises targeting major muscle groups (leg press, leg extension, leg curl, seated row, latissimus pull down, back extension, butterfly reverse and crunch) 4 weeks with 3 sets of 20 reps at 50–60% 1RM, then 4 weeks with 3 sets of 10–12 reps at 60–80% 1RM	X
Solheim et al., 2017^28^ (Norway) - RCT	46 various tumor types: Pancreas (*n* ​= ​20), lung (*n* ​= ​26) Surgery or CT or RT Age: 63.0 (54.5–68.0) (intervention), 59.0 (52.5–67.0) (control) Sex: 10W/15M (intervention), 10W/11M (control) Nutrition: Cachexia (Fearon): 100%	Resistance and endurance training 6 weeks 2 home-based endurance training of 30min / week ​+ ​3 home-based resistance training of 20 min / week	6 exercises (push-ups against the wall, overhead presses, biceps curls, squats, lunges and calf raise)	Nutritional (ONS ​+ ​counselling) Pharmacological (celexoxib)
Storck et al., 2020^29^ (swiss) - RCT	52 various tumor types: Colorectal (*n* ​= ​5), pancreas (*n* ​= ​9), renal (*n* ​= ​3), lung (*n* ​= ​22), breast (*n* ​= ​2), ovarian (*n* ​= ​3), prostate (*n* ​= ​6), Urothelial (*n* ​= ​2) NR Age: 62.0 ​± ​11.4 (intervention), 64.2 ​± ​9.2 (control) Sex: 11W/16M (intervention), 12W/13M (control) Nutrition: NRS: 1 (*n* ​= ​14), 2 (*n* ​= ​9), 3 (*n* ​= ​2), 4 (*n* ​= ​2), 5 (*n* ​= ​0) (intervention), 1 (*n* ​= ​14), 2 (*n* ​= ​7), 3 (*n* ​= ​0), 4 (*n* ​= ​4), 5 (*n* ​= ​0) (control)	Resistance and endurance training ​+ ​coordination 12 weeks 2 supervised mixed sessions of 90 and 60 min / week ​+ ​1 home-based session of 30min / week	Exercises with elastic bands, walking, cycling, circuit training 3 sets of 10–15 reps. RPE 4–6/10 (or 3/10 when chemotherapy the same day) Progression as appropriate	Nutritional (ONS ​+ ​counselling)
Uster et al., 2018^30^ (swiss) - RCT	58 various tumor types: Colorectal (*n* ​= ​16), oesophago-gastric (*n* ​= ​5), NSCLC (*n* ​= ​16), SCLC (*n* ​= ​4), pancreas (*n* ​= ​15), other (*n* ​= ​2) NR Age: 62.0 ​± ​9.3 (intervention), 64.0 ​± ​11.0 (control) Sex: 10W/19M (intervention), 8W/21M (control) Nutrition: NRS: 1 (*n* ​= ​11), 2 (*n* ​= ​11), 3 (*n* ​= ​3), 4 (*n* ​= ​2), 5 (*n* ​= ​2) (intervention), 1 (*n* ​= ​9), 2 (*n* ​= ​10), 3 (*n* ​= ​5), 4 (*n* ​= ​5), 5 (*n* ​= ​0)	Resistance training 12 weeks 2 supervised sessions of 60 min / week	5-6 exercises (leg press, leg flexion, pull down, abdominal trainer, bench press) 2 sets of 10 reps at 60–80% 1RM Progression as appropriate to realize 10 reps max	Nutritional (ONS ​+ ​counselling)
Wiskemann et al., 2019^31^ (Germany) - RCT	43 pancreatic cancer patients Surgery ​+ ​CT Age: 62.8 ​± ​6.4 (intervention supervised), 61.1 ​± ​8.7 (intervention home-based), 57.8 ​± ​8.2 (control) Sex: 4W/5M (intervention supervised), 8W/12M (intervention home-based), 7W/7M (control) Nutrition: Weight loss ≥ 10% during the last 6 months: 44.4% (intervention supervised), 65.0% (intervention home-based), 50.0% (control)	Resistance training 24 weeks 2 sessions of 60 min / week. Sessions supervised or home-based depending on group allocation.	5 exercises / session (supervised: Leg press, leg extension, leg curl, seated row, latissimus pull down, back extension, butterfly reverse, crunch; home-based: Resistance bands, dumbbels). 4 weeks with 1–2 set(s) of 20 reps at 50–60% 1RM. Then 3 sets of 8–12 reps at 60–80% 1RM. RPE 14–16/20 Progression as appropriate	X
Steindorf et al., 2019^32^ (Germany) - RCT	47 pancreatic cancer patients Surgery alone, or surgery ​+ ​CT, or CT alone Age: 62.8 ​± ​6.4 (intervention supervised), 61.0 ​± ​9.3 (intervention home-based), 58.7 ​± ​8.4 (control) Sex: 4W/5M (intervention supervised), 9W/12M (intervention home-based), 12W/8M (control) Nutrition: Weight loss ≥ 10% during the last 6 months: 44.4% (intervention supervised), 61.9% (intervention home-based), 47.1% (control)	Resistance training 24 weeks 2 sessions of 60min / week. Sessions supervised or home-based depending on group allocation.	8 exercises targeting major muscle groups 2-3 sets of 8–12 reps at 60–80% 1RM. RPE 14–16/20 Progression as appropriate	X
Fagevik Olsen et al., 2017^33^ (Sweden) - RCT	43 esophagus cancer patients Surgery, CT or RT neoadjuvant Age: 62.7 ​± ​10.7 (intervention), 62.3 ​± ​8.4 (control) Sex: 4W/16M (intervention), 4W/19M (control) Nutrition: Weight loss since symptoms: −5.8 ​± ​8.5% (intervention), −7.7 ​± ​7.3% (control) of body weight	Resistance training ​+ ​mobility 12 weeks Daily session. 1 week of supervised sessions then home-based sessions.	8-11 exercises to restore lung function, thoracic and spinal mobility, and the strength of spinal, shoulder and leg extensors. 10 reps per exercise Progression as appropriate	X
Arrieta et al., 2019^34^ (France) - RCT	300 various tumor types: Colorectal (*n* ​= ​35), breast (*n* ​= ​107), other (*n* ​= ​158) Surgery or CT or RT or HT or targeted therapy Age: 76.8 ​± ​5.1 (intervention), 76.6 ​± ​5.0 (control) Sex: 97W/53% (intervention), 83W/67M (control) Nutrition: MNA: Good nutrition (score > 11): *n* ​= ​54, at risk/poor nutrition (score ≤ 11): *n* ​= ​86 (intervention). Good nutrition (score > 11): *n* ​= ​49. At risk/poor nutrition (score ≤ 11): *n* = ​96 (control)	Resistance and endurance training ​+ ​balance ​+ ​flexibility ​+ ​proprioception 12 months 2 home-based resistance sessions / week. Recommendations to implement endurance training.	Exercises targeting major muscle groups (arm curls, hip abduction, hip adduction, squats …) 10 reps per exercise. Without and then with loads. Progression as appropriate	X
De lazzari et al., 2024^35^ (Germany) - RCT	31 various tumor types: Pancreas (*n* ​= ​19), biliary tract (*n* ​= ​12) CT Age: 61 (30–86) (intervention), 63 (37–74) (control) Sex: 9W/6M (intervention), 8W/8M (control) Nutrition: Body weight variation in 6 months: No weight loss (*n* ​= ​5), loss 0–2% (*n* ​= ​3), loss 2–5% (*n* ​= ​2), loss ≥ 5% (*n* ​= ​5) (intervention), No weight loss (*n* ​= ​9), loss 0–2% (*n* ​= ​0), loss 2–5% (*n* ​= ​1), loss ≥ 5% (*n* ​= ​6) (control)	Resistance training 8 weeks 1 supervised resistance session of 45 min / week ​+ ​2 home-based resistance sessions / week.	Exercises targeting major muscle groups (supervised: Leg press, bench press, latissimus pulldown, crunch and back extension; home-based: Body weight and resistance bands) 3 sets of 12 reps at 50% 1RM Progression as appropriate	X
Lonbro et al., 2013b^36^ (Denmark) – RCT	41 head and neck cancer patients RT Age: 55 ​± ​7 (intervention), 58 ​± ​7 (control) Sex: 5W/11M (intervention), 3W/17M (control) [error in article] Nutrition: Weight loss (in kg of pre-treatment body weight): 8.5 ​± ​5.7 (intervention), 7.4 ​± ​5.0 (control)	Resistance training ± 12 weeks (30 training) Supervision for 2–3 initial sessions and approximately 5 occasional supervisions.	7 exercises targeting major muscle groups (leg press, knee extension, hamstring curls, chest press, sit ups, back extensions and lateral pull down). 2 sets at a load corresponding to 15 RM Progression as appropriate of the load to reach 3 sets of 8 RM	X
Parent et al., 2025^37^ (France) – RCT	225 pancreatic cancer patients CT Age:64 (29–87) Sex: 93W/132M Nutrition: Low SMI (SMI < 41 cm^2^/m^2^ for women, < 43 cm^2^/m^2^ and < 53 cm^2^/m^2^ for men with BMI < 25.0 and ​≥ ​25.0 kg/m^2^, respectively): 46%	Resistance and endurance training 16 weeks 3-5 endurance or mixed sessions of 30 min / week. At least 2 sessions with resistance activities / week. Weekly remote supervision by an professional trainer and unsupervised sessions with a physical activity partner	Exercises targeting major muscle groups with elastic bands. 8 color-coded resistance level bands. Moderate intensity: Speak comfortably and 50% of 1RM. Progression as appropriate. 1 week with lower intensity activity could be planned every 4 weeks to prevent patient exhaustion.	X
Lonbro et al., 2013a^22^ (Denmark) – RCT	30 head and neck cancer patients RT Age: 56 (27–72) (exercise ​+ ​nutrition), 59 (40–68) (exercise) Sex: 5W/11M (exercise ​+ ​nutrition), 2W/12M (exercise) Nutrition: Weight loss (in kg of pre-treatment body weight): 10.5 ​± ​7.7 (exercise ​+ ​nutrition), 8.5 ​± ​7.5 (exercise)	Resistance training 12 weeks Unsupervised except for 2–3 initial sessions and approximately 5 occasional supervisions	7 exercises targeting major muscle groups (leg press, knee extension, harmstring curls, chest press, sit ups, back extensions and lateral pull down). 2 sets at a load corresponding to 15 RM Progression as appropriate of the load to reach 3 sets of 8 RM	Nutritional (on training days: Creatine (5 g) ​+ ​protein powder [30 g])
Naito et al., 2019^38^ (Japan) - NRCT	30 various tumor types: Pancreatic (*n* ​= ​6) and NSCLC (*n* ​= ​24) cancer patients CT Age: 75 (70–84) Sex: 10W/20M Nutrition: Fearon: Cachexie (*n* ​= ​12). Weight loss during the last 6 months: −3.0 ​± ​6.8% of body weight	Resistance ​+ ​counselling to increase daily steps 8 weeks Daily home-based sessions	3-5 exercises per day (calf raise, sit-to-stand, knee extension, knee raise, side leg raise) 3 sets of 10 reps. RPE 4/10. Progression as appropriate with enhancing number of exercises and add ankle weights.	Nutritional (ONS ​+ ​counselling) Physical activity promotive counselling
Parmar et al., 2017^39^ (Canada) - NRCT	374 various tumor types: Gastrointestinal (*n* ​= ​104), lung (*n* ​= ​137), hematological (*n* ​= ​48), breast (*n* ​= ​24), other (*n* ​= ​61) NR Age: 65.2 ​± ​13.3 Sex: 166W/208M Nutrition: Fearon: Pre-cachexia (*n* ​= ​64), cachexia (*n* ​= ​257), No cachexia (*n* ​= ​29), Unknown (*n* ​= ​24). Weight loss during the last 6 months: −10.2 ​± ​9.8% of body weight	Resistance and endurance training 12 weeks 2 supervised sessions / week. Exercise guide to add home-based sessions.	Individualized intensity.	Nutritional (counselling)
Avancini et al., 2023^40^ (Italy) - NRCT	12 various tumor types: Pancreatic (*n* ​= ​7), lung (*n* ​= ​5) CT, RT, surgery, immunotherapy or targeted therapy Age: 57.6 ​± ​7.4 Sex: 7W/5M Nutrition: Cachexia (EPCRC criteria): Yes (*n* ​= ​1) / No (*n* ​= ​11)	Resistance and endurance training 12 weeks 2 sessions of 60 min / week. Session could be supervised totally or partially with weekly phone call and worksheet	Exercises (squats, pulleys, push presses, sit-ups, calf-raises …) 2-3 sets of 8–12 reps at body weight or resistance bands. RPE 3–5/10. Progression as appropriate	X
Parker et al., 2019^41^ (USA) - NRCT	50 pancreatic cancer patients Surgery ​+ ​CT Age: 66 ​± ​8 Sex: 24W/26M Nutrition: Weight loss ≥ 3 kg in the last 6 months: *n* ​= ​37	Resistance and endurance training 16 weeks 2 home-based resistance sessions of 30min / week. 3 home-based endurance sessions of 20 min / week	8 exercises targeting major muscle groups with graded resistance tube or dumbbells 3 sets of 8–12 reps. RPE 12–13/20 Progression as appropriate	X
Sakamoto et al., 2024^42^ (Japan) - NRCT	1 esophagus cancer patient Surgery ​+ ​RT Age: 72 Sex: M Nutrition: Geriatric nutritional risk index: 87.4	Resistance and endurance (stationary ergometer) training ​+ ​inspiratory muscle training 12 weeks 5 supervised resistance and endurance sessions of 45 min / week. Twice daily for inspiratory muscle training	Exercises (shoulder and elbow flexion, squats, cuff raises, hip extension). 2 sets of 20 reps. RPE 13/20 Flexible	Nutritional (counselling)
Bland et al., 2021^43^ (Australia) - NRCT	162 various tumor types: Upper gastrointestinal (*n* ​= ​49), lung (*n* ​= ​38), colorectal (*n* ​= ​24), prostate (*n* ​= ​17), head and neck (*n* ​= ​8), other (*n* ​= ​26) NR Age: 67.2 ​± ​12.0 Sex: 68W/94M Nutrition: Fearon: Precachexia (*n* ​= ​7), cachexia (*n* ​= ​83), refractory cachexia (*n* ​= ​29), No cachexia (*n* ​= ​28), Unknown (*n* ​= ​15)	Resistance training Home-based session	NR	Nutritional (counselling) Pharmacological Physical activity education
Rosenberg et al., 2017^44^ (Germany) - NRCT	20 various tumor types: Renal (*n* ​= ​16), gastrointestinal (*n* ​= ​2), pancreas (*n* ​= ​1), liver (*n* ​= ​1) CT ​+ ​TKI substances Age: 65 ​± ​11 (intervention), 61 ​± ​6 (control) Sex: 2W/8M (intervention), 2W/8M (control) Nutrition: Weight loss > 10% of body weight in 4 weeks (*n* ​= ​2)	Resistance training 12 weeks 2 supervised sessions of 60 min / week	8 exercises (leg press, leg extension, leg curls, bench press, pulldown, seated row, butterfly, shoulder rotation). 1 set of 20 reps for warm-up and then 2 sets of 12 reps.	X
Argudo et al., 2021^23^ (Spain) - NRCT	33 esophagus cancer patients CT Age: 65 ​± ​12 Sex: 7W/26M Nutrition: Weight loss ≥ 10% in the last 6 months: *n* ​= ​13. PG-SGA: A (*n* ​= ​15), B (*n* ​= ​13), C (*n* ​= ​5)	High intensity interval training ​+ ​respiratory muscle training 5 weeks 5 supervised HIIT sessions of 60 min / week. Twice daily for respiratory muscle training	Warm-up and then 12x(1′ at 80% of Wpeak, r: 2′ at 40% of wpeak)	Nutritional (ONS ​+ ​counselling)
Luo et al., 2023^45^ (Australia) - NRCT	22 pancreatic cancer patients CT and/or RT, and/or surgery Age: 65.5 (53.5–71.0) Sex: 12W/10M Nutrition: Weight loss ≥ 10 kg in the past 12 months: *n* ​= ​10	Resistance and endurance training 12 weeks 2 supervised sessions of 60 min (30 min END ​+ ​30 min RES) / week	6-10 exercises (chest press, seated row, biceps curl, triceps extension, leg press, leg curl, leg extension, calf raise) 2-4 sets of 6–12 reps. Endurance training at 60–85% of HRmax. Progression as appropriate	Physical activity education to perform additional aerobic exercise
Parker et al., 2021^46^ (USA) - NRCT	97 pancreatic cancer patients Surgery ​+ ​CT Age: 67.7 ​± ​6.8 (intervention), 65.0 ​± ​8.9 (control) Sex: 14W/19M (intervention), 31W/33M (control) Nutrition: Sarcopenia: 21/33 (intervention), 40/64 (control)	Resistance and endurance training Until surgery 2 supervised resistance sessions of 30 min / week. Endurance home-based by bouts of 20min.	8 exercises (chest press, row, shoulder raises, biceps curls, triceps extensions, seated leg press, front and reverse hip lifts, abdominal rotations and seated crunches) with resistance tube 3 sets of 10–12 reps. RPE 12–13/20 Progression as appropriate	X
Glare et al., 2011^47^ (Australia) - NRCT	54 various tumor type: Lung (*n* ​= ​18), colorectal (*n* ​= ​9), upper GI (*n* ​= ​9), hepatopancreatobiliary (*n* ​= ​8), breast (*n* ​= ​4), prostate (*n* ​= ​2), other (*n* ​= ​4) CT only, CT ​+ ​RT, RT only, No treatment Age: 62 (24–85) Sex: 17W/33M Nutrition: Weight loss in the last 6 months: −10.2 (0–27)%	Resistance and endurance training 8 weeks 2 supervised or home-based sessions / week	NR	Nutritional (ONS ​+ ​counselling) Psychological support

EPCRC, European Palliative Care Research Collaborative; NRCT, Non-Randomized controlled trial; NSCLC, Non-small-cell lung cancer; SCLC, Small Cell Lung Cancer; CT, Chemotherapy; RT, Radiotherapy; W, Women; M, Men; n, number; 1RM, One repetition maximum; reps, repetitions; RPE, Rate of perceived exertion; MNA, Mini Nutritional Assessment; NRS, Nutrition Risk Score; PG-SGA, Patient-Generated Subjective Global Assessment; ONS, Oral Nutritional Supplements; GI, gastrointestinal; Wpeak, Peak power output. A: well-nourished; B: moderately undernourished or suspected; C: undernutrition.

Of the included studies, 13 focused solely on muscle resistance training, 12 combined resistance and endurance training, and 1 focused only on endurance training. The duration of the programs ranged from 5 weeks to 12 months, with an average of 13.86 ​± ​9.35 weeks. On average, the studies recommended that patients perform 3.10 ​± ​1.60 sessions per week (ranging from 2 to 7 sessions per week). Eleven studies offered a supervised exercise program, 7 offered a home-based program with telephone follow-up, and 8 studies provided a mix of supervised and home-based sessions. All studies, except for one that involved high-intensity training,^23^ targeted moderate-intensity exercise, which was assessed using the Borg scale, heart rate, or 1RM.

A total of 1936 patients participated (mean age: 63.80 ​± ​6.22 years; 822 [31.62%] women). Of these, 1357 patients underwent an exercise intervention (mean age: 63.29 ​± ​5.91 years; 529 [21.16%] women), while the remaining patients were in the control groups. The patients were undergoing treatment, primarily chemotherapy and radiotherapy, with or without surgery. Seven studies involved only patients with pancreatic cancer, four included patients with both pancreatic cancer and other types of tumors (lung [*n* ​= ​3], biliary tract [*n* ​= ​1]), three studies focused solely on patients with esophageal cancer, one combined patients with gastrointestinal and lung cancers, four studies included only patients with head and neck cancers, and eight studies included a variety of tumor types, with a high prevalence of digestive cancers.

Concerning the feasibility of exercise intervention, the eligibility rate was reported in 9 studies. Recruitment rate was reported in 22 studies, completion rate was reported in 24 studies and compliance with the intervention was reported in 23 studies. 19 studies assessed the safety of exercise reporting adverse events and serious adverse events.

Concerning physical outcomes, upper limb strength was assessed in 16 studies, lower limb strength was measured in 14 studies and aerobic capacity was measured in 12 studies. Functional capacity and Speed ability were assessed in 10 studies and coordination was measured in 8 studies. Balance and mobility were assessed in one study respectively. Concerning anthropometrics characteristics, weight was measured in 11 studies and BMI in 4. Few studies assessed body composition; only 7 studies measured lean mass and fat mass. Muscle mass was assessed in 5 studies. Physical activity and nutritional behaviors were assessed in 9 and 8 studies respectively.

Regarding psychological outcomes, QoL was assessed in 15 studies, cancer-related fatigue in 6 studies, anxiety and depression in 3 studies, pain in one study and other symptoms (nausea, vomiting, etc.) in 4 studies.

### Risk of bias in studies - results of individual studies

The quality assessment of the included studies is presented in Table 3. On average, the studies achieved a quality score of 5.19 ​± ​2.05/10, with an internal validity score of 3.38 ​± ​1.72/8 and a score of 1.76 ​± ​0.65/2 for result interpretability.

**Table 3:** Risk of bias assessment through PEDRo Scale.

Study	Q1	Q2	Q3	Q4	Q5	Q6	Q7	Q8	Q9	Q10	Q11	Intern validity (Q2:Q9)	Results interpretability (Q10:Q11)	Total
Capozzi 2016^24^	Y	Y	Y	Y	N	N	Y	N	Y	Y	Y	5	2	**7**
Grote 2018^25^	Y	Y	Y	Y	N	N	N	Y	Y	Y	Y	5	2	**7**
Hall 2021^26^	Y	Y	Y	Y	N	N	N	N	Y	Y	Y	4	1	**6**
Kamel 2020^27^	Y	Y	Y	Y	N	N	Y	Y	Y	Y	Y	6	2	**8**
Solheim 2017^28^	Y	Y	Y	N	N	N	N	Y	Y	Y	Y	4	2	**6**
Storck 2020^29^	Y	Y	Y	N	N	N	N	Y	Y	Y	Y	4	2	**6**
Uster 2018^30^	Y	Y	Y	Y	N	N	Y	Y	Y	Y	Y	6	2	**8**
Wiskemann 2019^31^	Y	Y	Y	Y	N	N	N	Y	Y	Y	Y	5	2	**7**
Steindorf 2019^32^	Y	Y	Y	Y	N	N	N	Y	Y	Y	Y	5	2	**7**
Fagevik olsen 2017^33^	Y	Y	Y	Y	N	N	N	N	Y	Y	Y	4	2	**6**
Arrieta 2019^34^	Y	Y	Y	Y	N	N	Y	Y	Y	Y	Y	6	2	**8**
De lazzari 2024^35^	Y	Y	Y	Y	N	N	N	N	Y	Y	Y	4	2	**6**
Lonbro, 2013b^36^	Y	Y	Y	N	N	N	Y	N	Y	Y	Y	4	2	**6**
Parent, 2025^37^	Y	Y	Y	Y	N	N	Y	Y	Y	Y	Y	5	2	**8**
Lonbro, 2013a^22^	Y	N	N	N	N	N	N	N	Y	Y	Y	1	2	**3**
Naito 2019^38^	Y	N	N	N	N	N	N	Y	Y	Y	Y	2	2	**4**
Parmar 2017^39^	Y	N	N	N	N	N	N	N	N	Y	Y	0	2	**2**
Avancini 2023^40^	Y	N	N	N	N	N	N	Y	Y	Y	Y	2	2	**4**
Parker 2019^41^	Y	N	N	N	N	N	N	Y	Y	N	N	2	0	**2**
Sakamoto 2024^42^	N	N	N	N	N	N	N	Y	Y	N	N	2	0	**2**
Bland 2021^43^	Y	N	N	N	N	N	N	Y	Y	Y	Y	2	2	**4**
Rosenberger 2017^44^	Y	N	N	Y	N	N	N	Y	Y	Y	Y	3	2	**5**
Argudo 2021^23^	Y	N	N	N	N	N	N	Y	Y	Y	Y	2	2	**4**
Luo 2023^45^	Y	N	N	N	N	N	N	N	Y	Y	Y	1	2	**3**
Parker 2021^46^	Y	N	N	N	N	N	Y	Y	N	Y	Y	2	2	**4**
Glare 2011^47^	Y	N	N	N	N	N	N	Y	Y	N	N	2	0	**2**

Y, Yes; N, No; Q1, Question 1; PEDro, Physiotherapy Evidence Database.

### Results of syntheses

#### Feasibility, compliance and safety

Feasibility was assessed through eligibility, recruitment and completion rates. Results are summarized in Table 4. Eleven studies reported rates of eligibility, which averaged 62.61% ​± ​22.80%. Among patients deemed eligible, the recruitment rate was 51.41% ​± ​26.32% (not reported [NR], *n* ​= ​4). Once enrolled in the program, the overall completion rate reached 75.33% ​± ​15.95% (NR, *n* ​= ​2). When comparing groups, completion rates were 77.35% ​± ​17.42% for the intervention group and 72.35% ​± ​13.83% for the control group.

**Table 4:** Feasibility and safety of exercise interventions.

	Eligibility rate (%)	Recruitment rate (%)	Completion rate (%)	Compliance rate (%)	Compliance: Supervised vs Home-based	Compliance: END vs RES sessions	Safety: SAEs (*n*^a^)	Safety: AEs (*n*^a^)
Mean ​± ​SD	62.6 ​± ​22.8	51.4 ​± ​26.3	All: 75.3 ​± ​15.9 IG: 77.3 ​± ​17.4 CG: 72.3 ​± ​13.8	All: 75.0 ​± ​13.9	Supervised: 73.0 ​± ​11.0 Home-based: 82.1 ​± ​9.2	END: 83.6 ​± ​2.3 RES: 43.3 ​± ​33.6	All: 31 [0–14] IG: 6.0 [0–6] CG: 7.0 [0–6]	All: 198.0 [0–93] IG: 85.0 [0–39] CG: 87.0 [0–54]

SD, Standard Deviation; IG, Intervention Group; CG, Control Group; END, Endurance training; RES, Resistance training; SAEs, Serious Adverse Events; AEs, Adverse Events.

a n is the total number of SAES or AES [0-14] indicates the minimum-maximum number of SAES per study.

Compliance to the exercise intervention averaged 75.02% ​± ​13.9% (NR, *n* ​= ​3). It was higher for endurance-based interventions (83.6% ​± ​2.3%) compared to muscle-strengthening interventions (43.3% ​± ​33.6%). Notably, home-based sessions demonstrated a slightly higher compliance rate (82.1% ​± ​9.2%) than supervised sessions (73.0% ​± ​11.0%).

Safety was assessed through adverse events and serious adverse events occurring during the exercise intervention. 19 studies evaluated the safety of exercise interventions by reporting the occurrence of moderate and serious adverse events. All studies concluded that exercise interventions were safe. Across all studies, 31 serious adverse events and 198 moderate adverse events were reported. These events were deemed unrelated to the intervention, as the intervention groups experienced 6 serious and 85 moderate adverse events, compared to 7 serious and 87 moderate events in the control groups.

#### Effects of exercise interventions

**Fig. 2:**
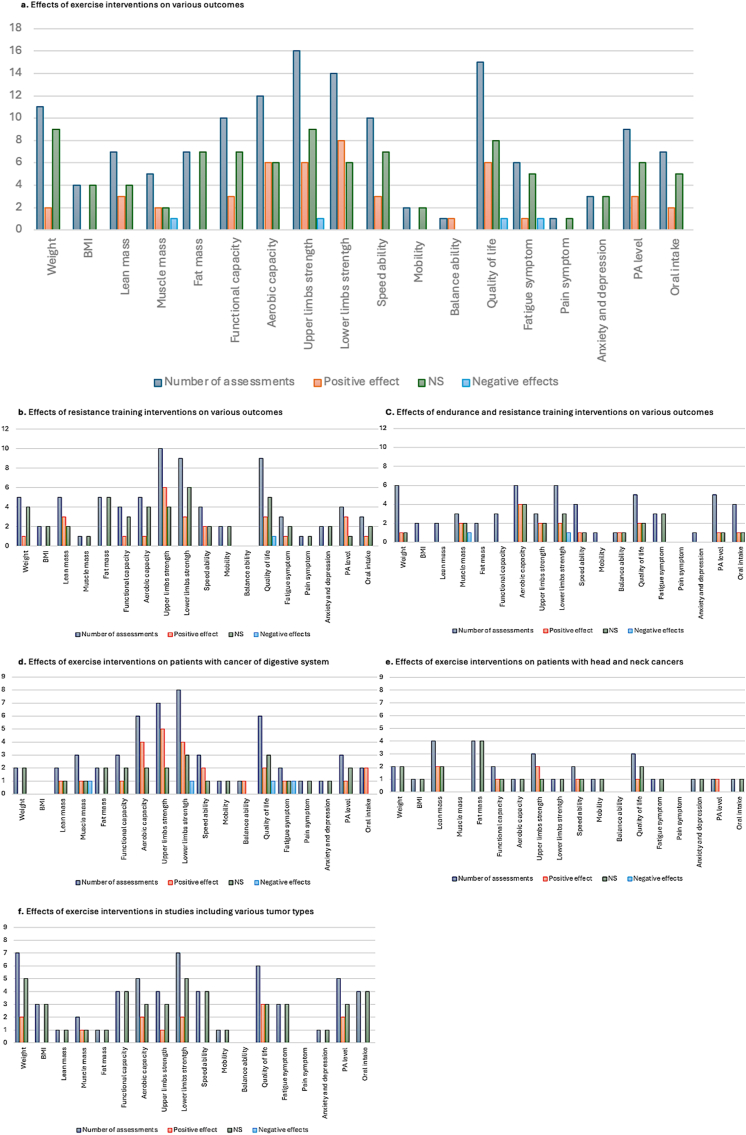
Effects of exercise on patients and differentiation regarding program characteristics and tumor type. 2a: Effects of exercise interventions. 2b: Effects of resistance training interventions. 2c: Effects of trainings combining resistance and endurance. 2d: Effects of exercise interventions in patients with cancer of digestive system, 2e: Effects of exercise interventions in patients with head and neck cancers. 2f: Effects of exercise interventions in studies including patients with various cancer. BMI, Body mass index; PA, Physical activity; NS, Non-significant.

The effects of the exercise interventions are detailed in Tables 5 and 6. Each outcome is presented alongside its measurement method and the associated p-value reflecting its evolution. The findings are categorized based on the type of intervention: all interventions combined, resistance-only and endurance-only. The results are summarized graphically in Fig. 2.

**Table 5:** Effects of time and exercise interventions in patients included in RCTs.

RCT	Outcomes (Measures) ​: Effects Group∗Time (*P*-value)
Capozzi 2016^24^	BMI: NS (*P* ​= ​0.698) LM (DEXA): NS (*P* ​= ​0.756) FM (DEXA): NS (*P* ​= ​0.741) Aerobic functional capacity (6MWT): NS (*P* ​= ​0.687) UL strength (handgrip): NS (*P* ​= ​0.086) LL strength (30s-STS): NS (*P* ​= ​0.079) Flexibility (dillion flexometer): NS (*P* ​= ​0.060) QoL (FACT-An, FACT HNSI-22): NS (*P* ​= ​0.751, *P* ​= ​0.503) Depression (CES-D): NS (*P* ​= ​0.519) Nutrition (PG-SGA): NS (*P* ​= ​0.846) PA level (Godin's leisure time exercise): IG ​> ​CG (*P* ​= ​0.009)
Grote 2018^25^	LM (BIA): NS (*P* ​= ​0.545) FM (BIA): NS (*P* ​= ​0.267) QoL (FAACT): NS (*P* ​= ​0.891) Fatigue (MFI): NS (all *P* ​> ​0.05)
Hall 2021^26^	Weight: NS (*P* ​= ​0.184) Speed (TUG): NS (*P* ​= ​0.767) Aerobic functional capacity (TMWT): NS (*P* ​= ​0.484) Functional capacity (LSA): NS (*P* ​= ​1.0) QoL (QLQ-C15, EQ-5DL, EQ-VAS): NS (*P* ​= ​0.846) Except emotional functioning IG ​> ​CG (*P* ​= ​0.006) Nutrition (abPG-SGA, AveS): NS (*P* ​= ​0.249, *P* ​= ​0.398) PA level (pedometer, Fitbit): NS (*P* ​= ​0.548)
Kamel 2020^27^	LM (BIA): Positive (*P* ​= ​0.001) FM (BIA): NS (*P* ​= ​0.55) UL & LL strength (dynamometer): Positive (*P* ​= ​0.001) LL strength (5-STS): Positive (*P* ​= ​0.001) Aerobic functional capacity (400 m walk): Positive (*P* ​= ​0.005) Speed (6m walk test): Positive (*P* ​= ​0.001)
Solheim 2017^28^	Weight: Positive (*P* ​< ​0.001) Muscle mass (CT-scan): Positive (*P* ​= ​0.030) UL strength (handgrip): NS (*P* ​= ​0.69) Aerobic functional capacity (6MWT): NS (*P* ​= ​0.32) Nutrition (aPG-SGA, AveS): NS (*P* ​= ​0.91, *P* ​= ​0.65) PA level (accelerometer, ActivPAL): NS (*P* ​= ​0.50) Fatigue (EN): NS (*P* ​= ​0.33)
Storck 2020^29^	Weight: NS (*P* ​> ​0.05) LM (BIA): NS (*P* ​> ​0.05) FM (BIA): NS (*P* ​> ​0.05) UL strength (handgrip): Positive at 3-month (*P* ​< ​0.001), NS at 6-month (*P* ​= ​0.284) LL strength (60s-STS): NS at 3 (*P* ​= ​0.824) and 6-month (*P* ​= ​0.218) Functional capacity (SPPB): NS at 3 (*P* ​= ​0.184) and 6-month (*P* ​= ​0.986) Speed (TUG): NS at 3 (*P* ​= ​0.065) and 6-month (*P* ​= ​0.735) QoL (QLQ-C30): NS (all *P* ​> ​0 0.05). Except dyspnea: Positive effect in IG (*P* ​= ​0.013) Nutrition (NRS-2002, 3-day food diary): NS at 3 (*P* ​= ​0.663) and 6-month (*P* ​= ​0.735) Fatigue (BFI): NS (*P* ​> ​0.05)
Uster 2018^30^	Weight: NS (*P* ​> ​0.05) UL strength (handgrip): NS (*P* ​> ​0.05) LL strength (1RM leg press, 30s-STS): NS (*P* ​> ​0.05) Aerobic functional capacity (6MWT): NS (*P* ​> ​0.05) QoL (QLQ-C30): NS (*P* ​> ​0.05). Except nausea and vomiting: Positive effect in IG (*P* ​< ​0.001) Nutrition (3-day food diary): Positive (*P* ​= ​0.01)
Wiskemann 2019^31^	UL strength (isokinetic dynamometer): Positive in IGS (*P* ​= ​0.02) LL strength (isokinetic dynamometer): Positive in IGS (*P* ​= ​0.01) and IGH (*P* ​= ​0.04) Aerobic functional capacity (6MWT): NS (*P* ​> ​0.05)
Steindorf 2019^32^	QoL (QLQ-C30, PAN26): Positive in global QoL, physical functioning, cognitive functioning, sleep problems (*P* ​< ​0.05) Fatigue (MFI): Positive in physical fatigue, reduced activity and motivation (*P* ​< ​0.05).
Fagevik olsen 2017^33^	UL strength (handgrip): NS (*P* ​> ​0.05) LL strength (10 stands-up from a chair, 10 heel rising): NS (*P* ​> ​0.05) Functional capacity (disability rating index): NS (*P* ​> ​0.05) Mobility (Thoracic flexion and extension): Positive (*P* ​< ​0.05) QoL (QLQ-C30): Positive in role functioning. Negative in global QoL and appetite loss (*P* ​< ​0.05) PA level (6 levels scale): NS (*P* ​> ​0.05) Pain (VAS): Positive (*P* ​< ​0.05)
Arrieta 2019^34^	Functional capacity (SPPB): NS (*P* ​= ​0.772) Speed (4m walk): NS (*P* ​= ​0.171) PA level (IPAQ): NS (*P* ​= ​0.856)
De lazzari 2024^35^	UL strength (handgrip): NS (right arm: *P* ​= ​00.502, left arm: *P* ​= ​0.429) LL strength (1RM leg press, 60s-STS): Positive (all *P* ​< ​0.03) QoL (QLQ-C30): NS. Except positive effects in fatigue (*P* ​= ​0.012) and constipation (*P* ​= ​0.045) symptoms PA level (BSA): Positive (*P* ​= ​0.019)
Lonbro, 2013b^36^	Lean body mass (DEXA): Positive (*P* ​= ​0.005) LL strength: Isometric KE: Positive (*P* ​= ​0.025), isometric KF: NS (*P* ​= ​0.182) Isokinetic KE: NS (*P* ​= ​0.366), isokinetic KF: Positive (*P* ​= ​0.005) Functional capacity (10 m gait speed, 30s-STS, maximal stair climbing, 30 s maximal arm curls): NS (all *P* ​> ​0.05) QoL (QLQ-C30): Positive on global health and cognitive function (*P* ​< ​0.05)
Parent, 2025^37^	Weight: NS (*P* ​> ​0.05) Muscle mass (DEXA): NS (*P* ​= ​0.89)

RCTs, randomized controlled trials; IG, Intervention Group; CG, Control Group; NS, Non-significant; BMI, Body Mass Index; DEXA, Dual-energy X-ray Absorptiometry; LM, Lean Mass; FM, Fat Mass; UL, Upper limbs; LL, Lower limbs; QoL, Quality of Life; PA, Physical Activity; 1RM, One Repetition maximum; 60s-STS, 60-s sit-to-stand test; 30s-STS, 30-s sit-to-stand test; 5-STS, 5 times sit-to-stand test; 6MWT, 6-min walk test; TMWT, Two-minutes walk test; TUG, Time-Up and Go test; m, meter; VAS, Visual Analog Scale; BFI, Brief Fatigue Inventory; FAACT, Functional Assessment of Anorexia/Cachexia Therapy; LSA, Life Space Assessment questionnaire; CES-D, Center for Epidemiologic Studies – Depression Scale; FACT-An, Functional Assessment of Cancer Therapy – Anemia; FACT HNSI-22, Functional Assessment of Cancer Therapy – Head/Neck Symptom Index-22; QLQ-C15, Quality of Life Questionnaire Core 15; EQ-5DL, European Quality of Life 5 Dimensions 5 Level; EQ-VAS, European Quality of Life Visual analogue scale; CT-Scan, Computed Tomography Scan; PG-SGA, Patient-Generated Subjective Global Assessment; BIA, Bio Impedance Analysis; EN, Numerical Rating Scale (Échelle Numérique [French]); NRS-2002, Nutrition Risk Screening 2002; SPPB, Short Physical Performance Battery; QLQ-C30, Core-30 Quality of Life Questionnaire; QLQ-PAN26, Quality of Life Questionnaire Pancreatic Cancer; MFI, Multidimensional Fatigue Inventory; BSA, Movement and Sport Activity Questionnaire (Bewegungs-und Sport-Aktivitats [Deutsch]); IPAQ, International Physical Activity Questionnaire; GNRI, Geriatric Nutritional Risk Index; MNA, Mini Nutritional Assessment; MVPA, Moderate to Vigorous Physical Activity; PW, Physical Well-being; EW, Emotional Well-being; FW, Functional Well-being; MVIC, Maximal voluntary isometric contraction; MIPT, Maximal isokinetic peak torque.

**Table 6:** Effects of exercise interventions in patients included in NRCTs.

NRCT	Outcomes (Measures): Effects of intervention (*P*-value)
Lonbro, 2013a^22^	Weight: NS (*P* ​> ​0.05) LM (DEXA): Positive (*P* ​< ​0.05) FM (DEXA): NS (*P* ​> ​0.05) LL strength (Maximal knee extensor [KE] and flexor [KF] strength [isokinetic dynamometry]): Positive (all *P* ​< ​0.05) Functional capacity (10m maximal gait speed, 30s-STS, maximal stair climbing, 30s maximal arm curls): Positive (all *P* ​< ​0.05) Nutrition (4 days diary): NR
Naito 2019^38^	BMI: NS (*P* ​> ​0.05) Muscle mass (CT scan): NS (*P* ​> ​0.05) UL strength (handgrip): Positive (*P* ​< ​0.05) LL strength (5-STS): Negative (*P* ​< ​0.05) Speed (5m gait speed): NS (*P* ​> ​0.05) Aerobic functional capacity (6MWT): NS (*P* ​> ​0.05) Nutrition (MNA, 2-day diet diary): NS (*P* ​> ​0.05) PA level (accelerometer): Positive in daily steps and time spent in MVPA (*P* ​< ​0.05)
Parmar 2017^39^	Weight: NS (*P* ​> ​0.05) QoL (FAACT): Positive (*P* ​< ​0.01)
Avancini 2023^40^	Weight: NS (*P* ​= ​0.436) BMI: NS (*P* ​= ​0.406) UL strength (handgrip): NS (right arm: *P* ​= ​0.387, left arm: *P* ​= ​0.494) LL strength (leg press test): NS (*P* ​= ​0.098) Aerobic functional capacity (6MWT): Positive (*P* ​= ​0.021) Flexibility (Back screatch test): NS (all *P* ​> ​0.05) QoL (QLQ-C30): Positive in emotional (*P* ​= ​0.004) and social functioning (*P* ​= ​0.003) PA level (Godin's leisure time exercise questionnaire): Positive in moderate PA (*P* ​= ​0.002)
Parker 2019^41^	PA level (accelerometer [ActiGraph GT3X+]): NS (*P* ​> ​0.05) PA level (IPAQ-SF): NS (*P* ​= ​0.08) PA level (PA diary): NS (*P* ​> ​0.05)
Sakamoto 2024^42^	Muscle mass (BIA): Negative UL strength (handgrip): Negative LL strength (5-STS): Positive Aerobic functional capacity (6MWT): Positive Speed (10 m gait speed): NS Functional capacity (SPPB): NS Nutrition (GNRI): Positive
Bland 2021^43^	Weight: NS (*P* ​= ​0.904) UL strength (handgrip): NS (*P* ​= ​0.734) LL strength (30s-STS): NS (*P* ​= ​0.133) QoL (QLQ-c15-PAL, FAACT): QLQ-C15-PAL: Positive in overall QoL, global health status, physical and emotional functioning, fatigue, pain, Nausea/Vomiting, dyspnea, insomnia, appetite loss symptoms (all *P* ​< ​0.05). FAACT: Positive in PW, EW, FW, anorexia/cachexia symptoms (all *P* ​< ​0.05)
Rosenberger 2017^44^	Weight: Positive (*P* ​= ​0.05) UL strength (elbow flexors strength (isokinetic dynamometer)): MVIC: NS (*P* ​= ​0.286). MIPT: NS (*P* ​= ​0.598) LL strength (Knee extensors strength (isokinetic dynamometer)): MVIC: Positive (*P* ​= ​0.005). MIPT: NS (*P* ​= ​0.083) Functional capacity (CPET): NS of VO_2_peak (*P* ​= ​0.459) and VT (*P* ​= ​0.417) QoL (QLQ-C30): NS (all *P* ​> ​0.05) Fatigue (MFI): NS (all *P* ​> ​0.05) Depression (CES-D): NS (*P* ​= ​0.384)
Argudo 2021^23^	UL strength (handgrip): NS (*P* ​> ​0.05) LL strength (isometric dynamometer): NS (*P* ​> ​0.05) Aerobic functional capacity (6MWT): Positive (*P* ​< ​0.05) Functional capacity (CPET): Positive in VO_2_peak, wpeak, VEpeak (*P* ​< ​0.05) QoL (QLQ-C30): Positive in social and role functioning, appetite loss and fatigue symptoms (*P* ​< ​0.05)
Luo 2023^45^	LM (DXA): NS (*P* ​= ​0.465) FM (DXA): NS (*P* ​= ​0.148) UL strength (1RM chest press and seated row): NS (*P* ​= ​0.496) and positive (*P* ​= ​0.036) LL strength (1RM leg press, 5-STS): Positive (*P* ​= ​0.008 and *P* ​= ​0.014) Aerobic functional capacity (400 m walk): Positive (*P* ​= ​0.009) Speed (6m fast pace walk): Positive (*P* ​= ​0.042) Static balance (NeuroCom sensory organization test): Positive (*P* ​= ​0.042) QoL (SF-36, FACT-hep): NS (all *P* ​> ​0.05) Fatigue (pittsburgh sleep quality index, FACIT-F): Negative in sleep quality (*P* ​= ​0.009) and NS in FACIT-F (*P* ​= ​0.959) Anxiety (BSI-18): NS (all *P* ​> ​0.05)
Parker 2021^46^	BMI: NS (*P* ​= ​0.30) SMI (CT scan): Positive (*P* ​= ​0.03) SMD (CT scan): NS (*P* ​= ​0.20)
Glare 2011^47^	Weight: NS UL strength (handgrip): Positive Aerobic functional capacity (6MWT): Positive Functional capacity (KPS): NS Symptoms (ESAS): Positive in Breathless, nausea, drowsiness and appetite

NRCTs, non-randomized controlled trials; IG, Intervention Group; CG, Control Group; NS, Non-significant; BMI, Body Mass Index; DEXA, Dual-energy X-ray Absorptiometry; LM, Lean Mass; FM, Fat Mass; UL, Upper limbs; LL, Lower limbs; QoL, Quality of Life; PA, Physical Activity; 1RM, One Repetition maximum; 60s-STS, 60-s sit-to-stand test; 30s-STS, 30-s sit-to-stand test; 5-STS, 5 times sit-to-stand test; 6MWT, 6-min walk test; TMWT, Two-minutes walk test; TUG, Time-Up and Go test; m, meter; VAS, Visual Analog Scale; BFI, Brief Fatigue Inventory; FAACT, Functional Assessment of Anorexia/Cachexia Therapy; LSA, Life Space Assessment questionnaire; CES-D, Center for Epidemiologic Studies – Depression Scale; FACT-An, Functional Assessment of Cancer Therapy – Anemia; FACT HNSI-22, Functional Assessment of Cancer Therapy – Head/Neck Symptom Index-22; QLQ-C15, Quality of Life Questionnaire Core 15; EQ-5DL, European Quality of Life 5 Dimensions 5 Level; EQ-VAS, European Quality of Life Visual analogue scale; CT-Scan, Computed Tomography Scan; PG-SGA, Patient-Generated Subjective Global Assessment; BIA, Bio Impedance Analysis; EN, Numerical Rating Scale [Échelle Numérique (French]); NRS-2002, Nutrition Risk Screening 2002; SPPB, Short Physical Performance Battery; QLQ-C30, Core-30 Quality of Life Questionnaire; QLQ-PAN26, Quality of Life Questionnaire Pancreatic Cancer; MFI, Multidimensional Fatigue Inventory; IPAQ, International Physical Activity Questionnaire; GNRI, Geriatric Nutritional Risk Index; MNA, Mini Nutritional Assessment; MVPA, Moderate to Vigorous Physical Activity; PW, Physical Well-being; EW, Emotional Well-being; FW, Functional Well-being; MVIC, Maximal voluntary isometric contraction; MIPT, Maximal isokinetic peak torque; SMI, Skeletal muscle index; SMD, Skeletal muscle radiodensity; ESAS, Edmonton Symptom Assessment System; KPS, Karnofsky Performance Scale; BSI-18, Brief Symptom Inventory-18 questionnaire; SF-36, Short-Form-36 questionnaire; FACT-Hep, Functional Assessment of Cancer Therapy–Hepatobiliary; Wpeak: Peak power output.

Overall, exercise interventions did not result in adverse outcomes. For the most assessed parameters—functional and aerobic capacity, muscle strength, body weight, QoL, and physical activity levels—the findings ranged from non-significant to positive changes. Results are synthetized in Fig. 2A (Blue: number of assessments, Orange: number of significant positive effects, Green: number of non-significant effects, Blue: number of significant negative effects).

When comparing randomized controlled trials (RCTs) with non-randomized controlled trials (NRCTs), NRCTs reported more positive effects, whereas RCTs more frequently found non-significant changes relative to control groups. Detailed results are provided in the Supplementary Material 2.

#### Exercise characteristics

Additional analyses examined the characteristics of the exercise programs based on the FITTS framework: Frequency, intensity, temporality, type and supervision. Comparisons related to program intensity were limited as 20/26 interventions implemented moderate intensity. Two interventions individualized intensity^34^^,^^39^ and one study proposed high intensity interval training HIIT.^23^ Three studies did not report the intensity of the sessions. Detailed results are available in the Supplementary Material 3.

##### Exercise type

In terms of changes in muscle mass and lean body mass, among the six studies involving resistance training alone, three reported significant increases, while the other three observed no significant change.

Of the six interventions combining resistance and endurance training, two demonstrated significant gains, three showed non-significant changes, and one reported a significant decrease. The single endurance-only intervention did not assess this parameter. These findings suggest a potential influence of exercise type on muscle or lean mass, with resistance training possibly yielding more favorable outcomes; however, this conclusion remains tentative.

For muscle strength, the 19 assessments from resistance training programs showed nine significant improvements and ten non-significant results. Among the nine assessments from combined resistance and aerobic training, five indicated significant gains, three showed no significant change, and one reported a significant decrease in strength. The two evaluations of endurance-only programs found no significant change in muscle strength. As expected, interventions that included endurance training were associated with greater improvements in functional and aerobic capacity compared to resistance-only programs.

Regarding PROs, such as QoL and oral intake, improvements seemed improving in the same way regardless of the type of exercise proposed. For PA level, resistance training programs led to improvements in three out of four evaluations, while combined resistance and endurance trainings resulted in two out of five improvements.

##### Exercise frequency

Twelve studies implemented programs with more than two sessions per week, and another twelve included two sessions per week. Two studies did not report exercise frequency. Among studies evaluating muscle strength, interventions with one to two sessions per week led to significant improvements in eight out of twelve assessments, while four showed no significant effect. In contrast, programs with more than two sessions per week led to five significant improvements, nine non-significant results, and one significant decrease across fifteen assessments. Similar trends were observed for QoL. No consistent patterns emerged regarding aerobic and functional capacity, oral intake, or physical activity level. These findings suggest that increasing frequency beyond two sessions per week may not enhance, and could potentially reduce, muscular benefits.

##### Exercise duration

The majority of studies (17 out of 26) implemented interventions lasting more than eight weeks. Two studies used flexible durations (i.e., during radiotherapy or until surgery),^25^^,^^46^ and one did not report duration.^43^ No clear trends emerged concerning intervention duration for outcomes related to muscle mass, aerobic or functional capacity, physical activity level, or QoL. In terms of muscle strength, the eight assessments from short-duration programs (≤ 8 weeks) reported three significant improvements and five non-significant effects. The two shortest programs (5 and 6 weeks) reported no significant changes.^23^^,^^28^ In contrast, longer interventions (> 8 weeks) led to eleven significant improvements, eight non-significant findings, and one decrease in muscle strength. These results suggest a potential advantage of longer interventions for improving muscle strength.

##### Exercise supervision

Eleven interventions were fully supervised, eight combined supervised and home-based components, and seven were entirely unsupervised. Due to the limited number of assessments per category (3–5), no conclusions could be drawn regarding supervision effects on muscle mass, lean body mass, or QoL. Similarly, all types of programs showed comparable effects on aerobic and functional capacities. For muscle strength, supervised interventions (14 assessments) resulted in nine significant improvements, four non-significant outcomes, and one significant decline. Mixed programs (11 assessments) produced five significant and six non-significant outcomes. Unsupervised interventions (five assessments) showed no significant improvements. These results suggest that supervised or partially supervised programs may yield greater benefits for muscle strength. Notably, improvements in oral intake were observed only in fully supervised interventions. For physical activity level, mixed programs appeared to be the most beneficial.

#### Effects of patient characteristics on response to exercise

Additional analyses examined patient responses to exercise based on tumor type, age, and sex. Changes in muscle and lean body mass were generally similar across tumor types. However, studies involving younger patients and a higher proportion of women reported more favorable outcomes for this parameter. QoL improvements also appeared more pronounced among female participants. Regarding muscle strength, greater benefits were observed in patients with digestive cancers and in studies with a higher proportion of women. Patients with head and neck cancers exhibited the smallest gains in aerobic and functional capacities. Notably, improvements in nutritional intake were only seen in patients with digestive cancers. Additional results are available in the Supplementary Material 3.

## Discussion

### Main findings

Our systematic review aimed to summarize current evidence on the effects of exercise interventions in patients with digestive and head and neck cancers, populations with undernutrition, anorexia, sarcopenia, and/or cachexia (i.e. cachexia prevalence exceeds 70% in digestive cancer and head and neck cancer). We analyzed 26 trials published between 2011 and 2024. Overall, exercise interventions were deemed safe and feasible in these patients and could lead to beneficial effects on muscle mass, functional and aerobic capacity, lower and upper limbs strength and QoL.

A significant feasibility challenge is patient recruitment, which often spans years, with many studies failing to meet target enrollment (e.g., Storck et al.,^29^ 53/88 participants; Steindorf et al.,^32^ 61/201; Rosenberger et al.,^44^ took 34 months to recruit their 20 participants). Our findings align with prior studies indicating that patients with cancer cachexia have the physiological capacity to benefit from exercise.^48^ However, initiating and sustaining participation remains challenging.^2^^,^^49^ Patients in the pre-cachexia phase may deprioritize exercise, as early muscle loss is overshadowed by more immediate concerns like cardiotoxicity or comorbidities.^50^

Recruitment barriers include limited healthcare provider recommendations—only 34.3% of clinicians prescribe exercise for cancer cachexia, with 23.7% expressing low confidence in managing it.^12^ Additionally, misconceptions among patients, caregivers, and clinicians, such as the belief that exercise exacerbates weight loss, further hinder participation. Addressing such misconceptions is critical to fostering engagement.^51^^,^^52^ A final barrier to commitment to an exercise intervention may be the constraints associated with travel, and the associate cost and timetables.^53^, ^54^, ^55^, ^56^, ^57^ In response to this, De Lazzari et al.^35^ proposed a protocol comprising one supervised session and two home-based sessions, which resulted in a high recruitment rate. This type of solution shows promise for future implementation.^58^

Completion rates exceeded 70% for both intervention and control groups, with higher attrition in control groups potentially linked to a lack of perceived benefits without receiving the intervention or greater health complications. Intervention compliance averaged 75.02% ​± ​13.9%, with endurance programs showing higher adherence (83.6 ​± ​2.3%) compared to resistance programs (43.3 ​± ​33.6%). Compliance also varied with patient demographics, favoring younger patients and those with fewer treatment side effects.^24^^,^^47^ These results are in line with those found in women with breast cancer^59^ or in elderly patients.^60^ Thus, the more deconditioned and at-risk patients are, the greater the challenge of initiating exercise becomes. One solution could be that proposed by Lonbro,^22^ which encourages patients to carry out 30 muscle-strengthening sessions over 12 weeks. This strategy allows patients to manage their progress in the program in the light of their state of health and leads to 97% compliance with the program. On average, patients take 13 (12–20) weeks to complete the 30 sessions.

While the benefits of exercise are well established in early-stage cancers such as colorectal, breast, and prostate,^61^ evidence remains limited for patients with cancer cachexia. Among the 16 studies assessing upper-limb strength, only six reported improvements, nine found no effect, and one reported a decline. These inconsistent findings may partly result from measurement bias, particularly in patients affected by hand-foot syndrome.^9^ A similar pattern is observed for muscle mass and lean body mass, with fewer than half of the studies reporting significant increases.

These mixed outcomes are consistent with prior literature. While some studies suggest grip strength correlates with muscle mass,^62^ more recent evidence challenges this association and points out that grip strength is not consistently related to other physical function measures, except for gait speed.^63^ Lower-limb strength, on the other hand, improved in over half the studies. This supports the notion that lower limbs, which are more consistently engaged in daily life and across all interventions, may better reflect muscular adaptations than upper-limb measures.

Regarding aerobic capacity, six studies showed significant improvements, while six did not. These results are still encouraging given the timing of interventions, often during anticancer treatments, where the goal is to attenuate deconditioning. In cachectic patients, stabilizing functional parameters may itself suggest a slowing of disease progression or compensation for muscle and mitochondrial loss.

Another factor contributing to these equivocal results is limited sample size. Most included studies were feasibility trials, often underpowered to detect changes in physical parameters. Even when powered, calculations were typically based on QoL endpoints rather than physiological outcomes.^26^^,^^28^ Regarding QoL, improvements were reported in 40% of studies, similar to the 38% reported by Cheung et al.^13^ As a multidimensional construct, QoL may show improvements in some domains but deterioration in others. For example, Parker et al.^41^ reported improved role functioning but worsening appetite loss and global scores. Similarly, Hall et al.^26^ found improvements only in emotional well-being. Our findings are therefore consistent with existing literature in cachexia.

Compared to patients with localized cancers,^61^ individuals with cachexia may derive fewer benefits from exercise due to disease burden, visible body changes, and daily activity limitations.^64^ Moreover, disease progression during the intervention may blunt any gains. However, this hypothesis is nuanced by findings from randomized controlled trials. Of the 13 RCTs included, many reported similar outcomes between intervention and control groups. This could reflect compensatory behavior in both arms: patients engaging in supervised exercise may reduce other forms of activity, maintaining an overall stable physical activity level,^28^ while control group participants may increase their activity in anticipation of physical testing—a possible contamination effect.^29^

Only two assessments reported negative effects on upper-limb muscle mass and strength. Both were based on a case study involving a 72-year-old man undergoing adjuvant therapy for esophageal cancer with multiple risk factors.^42^ Advanced age, male sex, and preoperative radio-chemotherapy are known risk factors for severe muscle loss during treatment and likely explain the adverse outcomes.

Patient characteristics also appear to influence the response to exercise. Studies with higher proportions of women tended to report more favorable outcomes in muscle mass, strength, and QoL. This may be due to sex-based physiological differences and potentially greater trainability. This result is consistent with the literature reporting greater muscle mass loss in men.^65^^,^^66^ Sociological factors may also play a role, as women tend to engage in less regular physical activity, possibly resulting in a greater relative response when exposed to structured training.

In addition to patient-related factors, intervention design significantly influenced outcomes. While gains in muscle mass and strength were similar across resistance-only and combined (resistance ​+ ​endurance) programs, improvements in aerobic and functional capacity were more frequent in combined protocols. Supervised interventions yielded the greatest improvements across all dimensions—strength, aerobic fitness, speed, and nutritional intake—followed by partially supervised formats and then home-based programs. These findings align with Cheung et al.,^13^ but our review encompasses a broader dataset. These differences may be explained by better load regulation and progression in supervised settings.

Finally, exercise should be considered as one component of a multimodal approach to managing cancer cachexia. Lonbro et al.^22^ demonstrated that combining exercise with protein and creatine supplementation led to superior outcomes compared to exercise alone. This underscores the importance of integrated strategies targeting both physical and nutritional aspects.

In summary, exercise interventions appear effective in slowing disease-related decline, enhancing physical function, and improving QoL in patients with digestive and head and neck cancers. However, outcomes are influenced by both patient characteristics and intervention design. Supervised, combined programs, tailored to individual needs and supplemented with nutritional support, may offer the most promising results.

### Strengths and limitations

Our review includes a substantial number of studies (*n* ​= ​26) and patients (*n* ​= ​1936), offering insights into exercise effects in this under-researched population. By expanding inclusion criteria to encompass cachexia-related terms (e.g., sarcopenia, malnutrition), we address diagnostic challenges and enhance generalizability. Eight studies were of high methodological quality based on the PEDro scale. The low internal validity can be attributed primarily to the absence of control groups in non-randomized controlled trials (NRCTs). In randomized controlled trials (RCTs), the lack of blinding of patients, therapists, and evaluators further undermines validity. High dropout rates during interventions and recruitment challenges also reduced statistical power, limiting the robustness of the reported results. The relatively high interpretability score reflects the clarity of statistical reporting across most studies. However, this was an exception for the studies by Glare et al.,^42^ Bland et al.,^43^ and Sakamoto et al.,^47^ which lacked detailed statistical reporting. In a word, the methodological quality of included studies is constrained by limited randomization, lack of blinding, and high dropout rates, which may introduce motivational and observer biases.

In addition, the 1936 patients included in our review were highly heterogeneous in terms of selection criteria (e.g., Patient-Generated Subjective Global Assessment [PG-SGA] vs. Fearon criteria), tumor type, age, sex, and concurrent treatments. This diversity likely resulted in the inclusion of patients with varying degrees of malnutrition and energy imbalance. Our additional analyses suggest that such heterogeneity may contribute to differences in response to exercise interventions. However, further stratified research is needed to better understand which patient profiles benefit most. However, heterogeneity in patient profiles, nutritional supports, treatment type and temporality, intervention protocols, and outcome measures limits the detection of specific effects. These elements reflect real-life contexts and can help yield representative and generalizable results for a broader population of patients affected by malnutrition, cachexia, anorexia, and sarcopenia.^21^^,^^67^

The voluntary nature of participation introduces bias, as healthier patients are more likely to engage in and complete interventions, while those in poorer health are often lost to follow-up.^39^ Similarly, in Storck et al.'s study,^29^ baseline SPPB scores are already high, and patients are in good physical condition. This highlights the need to find solutions to reach patients who are furthest from engaging in physical activity, as they may potentially derive the greatest benefit.^68^ Thus, our results are representative of the effects of exercise in volunteer patients who have a certain affinity for exercise and a minimum level of physical fitness. Moreover, most included studies performed per-protocol analyses, which may introduce bias by excluding patients who dropped out of the intervention. This can lead to an overestimation of exercise effects, as outcomes for non-completers—who may have experienced deterioration—are not considered.^69^ Similarly, Glare et al.^47^ highlighted that the most compliant patients tend to have less weight loss, fewer nutritional impairments, lower inflammation (e.g., CRP levels), and better baseline physical condition. As a result, pre-post intervention changes may primarily reflect the effects of exercise in a healthier subset of patients.

### Implications for practice and research

Healthcare professionals should encourage both endurance and resistance exercise in patients with cachexia, as these interventions may help mitigate disease progression and improve muscle mass, strength, aerobic capacity, QoL, and nutritional intake. Supervised programs are preferable, but hybrid models—including home-based or remotely monitored sessions—can enhance accessibility and adherence.

Given the importance of preserving energy balance in this population, investigating the dose–response relationship of exercise could help optimize intervention strategies. Furthermore, to maximize muscle mass gains while minimizing energy expenditure, eccentric resistance training may offer advantages. A pioneering study in women with breast cancer suggests that eccentric training can be particularly effective in this regard.^70^ Future studies should compare the effects of eccentric versus concentric resistance training in severely malnourished patients to refine exercise recommendations.

Future research should prioritize larger, rigorously designed trials to assess exercise effects on cachexia-specific outcomes, such as muscle mass and strength. Studies should also explore real-life implementation strategies to optimize patient engagement and adherence while accommodating medical and personal constraints.

## Conclusions

Exercise interventions are safe, feasible, and potentially beneficial for patients with digestive or head and neck cancers affected by cachexia, undernutrition, anorexia, or sarcopenia. Despite common recruitment challenges, high rates of compliance and program completion suggest that exercise can help stabilize or improve both physical and psychological health—even in the context of disease progression.

The effectiveness of exercise appears to depend on both patient-specific characteristics and the design of the intervention. Preserving muscle mass and strength is particularly important, as it may reduce fatigue, enhance treatment tolerance, and improve overall survival in this vulnerable population.

## CRediT authorship contribution statement

**RP** and **AR** designed research; **RP** and **AR** did selection process and study quality assessment; **RP** analyzed data; **RP** and **AR** wrote the paper. **AL** supervised the project. All authors read and approved the final manuscript.

## Ethics statement

Not applicable.

## Data availability statement

The authors confirm that the data supporting the findings of this study are available within the article and its supplementary materials.

## Declaration of generative AI and AI-assisted technologies in the writing process

During the preparation of this work, the authors used Deepl.com and ChatGPT in order to check English spelling.

## Funding

This work was supported by the University of Rennes 2. The funders had no role in considering the study design or in the collection, analysis, interpretation of data, writing of the report, or decision to submit the article for publication.

## Declaration of competing interest

The authors declare no conflict of interest.
