# Supplementary material for: The role of aerobic and resistance exercise for cancer cachexia management – A systematic scoping review
Source: Asia Pac J Oncol Nurs. 2025 Jun 30;12:100748. doi: 10.1016/j.apjon.2025.100748 (PMC12274876; doi:10.1016/j.apjon.2025.100748)
Supplement: Multimedia component 1 [file mmc1.docx]

Supplementary material 1

Databases, registers, websites used. Date of last database search.

Prospero: no systematic reviews currently available on PROSPERO as of 23/02/2025. One systematic review looks at the effects of PA on the development of cachexia, focusing on body composition and BMI (Horawski et al., 2023).

Three search databases were explored: PubMed, Google Scholar, and EBSCOhost (which includes MEDLINE, Academic Search Premier, SPORTDiscus with Full Text, Psychology and Behavioral Sciences Collection, and eBook Collection).

Keywords were defined using the PICTOS method, and MeSH terms were identified on HeTOP (NLM).

The search equations are available in Supplementary Material 1. We verified that no systematic reviews on this topic were in progress using the PROSPERO identification registry.

As of February 23, 2025, no systematic reviews have been conducted on the effects of exercise interventions in patients with digestive cancers at risk of cachexia or who are cachectic.

Research question :

PICOTS

|  | Population | Intervention | Comparaison | Outcomes | Time | Study design |
| --- | --- | --- | --- | --- | --- | --- |
| Key words | Malnourished cancer patients  Cancer patients with cachexia or sarcopenia  Pancreatic cancer patients  Gastro-esophageal cancer patients | Exercise, Physical Activity, Training,  Rehabilitation | Themselves or usual care | Quality of life, fatigue, depression  Physical fitness : endurance, strength, physical activity level  Body composition and stature : weight, BMI, lean mass, muscle mass, fat mass | More than 2 weeks | RCT and NRCT |
| MeSH Terms (HeTOP) | Neoplasms  Cancer of digestive system  Cancer of head and neck  Cachexia  Sarcopenia  Malnutrition | Exercise  Rehabilitation |  |  |  |  |

**Research equations: 23/02/2025**

(« Malnourished cancer patients » OR « Pancreatic cancer patients » OR “Digestive cancer patients” OR “Gastro-esophageal cancer patients” OR “Head and Neck” OR “Cancer cachexia” OR “Cancer undernutrition” OR “Cancer sarcopenia”) AND (“Exercise” OR “Physical Activity” OR “Training” OR “Rehabilitation”)

**PubMed:**

Search: **Search: (« Malnourished cancer patients » OR « Pancreatic cancer patients » OR "Digestive cancer patients" OR "Gastro-esophageal cancer patients" OR “Head and neck cancer patients” OR "Cancer cachexia" OR "Cancer undernutrition" OR "Cancer Sarcopenia") AND ("Exercise" OR "Physical Activity" OR "Training" OR "Rehabilitation") Filters: Clinical Trial, Randomized Controlled Trial**

(("Malnourished cancer patients"[All Fields] OR "Pancreatic cancer patients"[All Fields] OR "Digestive cancer patients"[All Fields] OR "Gastro-esophageal cancer patients"[All Fields] OR "Cancer cachexia"[All Fields] OR (("cancer s"[All Fields] OR "cancerated"[All Fields] OR "canceration"[All Fields] OR "cancerization"[All Fields] OR "cancerized"[All Fields] OR "cancerous"[All Fields] OR "neoplasms"[MeSH Terms] OR "neoplasms"[All Fields] OR "cancer"[All Fields] OR "cancers"[All Fields]) AND ("malnutrition"[MeSH Terms] OR "malnutrition"[All Fields] OR "undernutrition"[All Fields] OR "undernutritional"[All Fields])) OR "Cancer Sarcopenia"[All Fields]) AND ("Exercise"[All Fields] OR "Physical Activity"[All Fields] OR "Training"[All Fields] OR "Rehabilitation"[All Fields])) AND (clinicaltrial[Filter] OR randomizedcontrolledtrial[Filter])

**Google Scholar:**

Cancer patients malnutrition cachexia sarcopenia pancreatic digestive esophageal exercise physical activity training exercise OR physical OR activity OR intervention OR training OR rehabilitation

**SPORTDiscus / EBSCOhost:** rassemble plusieurs bases de données : MEDLINE, Academic Search Premier, SPORTDiscus with Full Text, Psychology and Behavioral Sciences Collection, eBook Collection (EBSCOhost)

Équation :

( digestive or pancreatic or intestinal or gastro or esophageal ) AND TX ( Cachexia or malnutrition or undernutrition or sarcopenia ) AND TI ( Exercise or physical activity or training or rehabilitation ) AND TI Cancer
