# Supplementary material for: The role of aerobic and resistance exercise for cancer cachexia management – A systematic scoping review
Source: Asia Pac J Oncol Nurs. 2025 Jun 30;12:100748. doi: 10.1016/j.apjon.2025.100748 (PMC12274876; doi:10.1016/j.apjon.2025.100748)
Supplement: Multimedia component 3 [file mmc3.docx]

Outcomes / Exercise program characteristics

Muscle Mass

|  | *Number of assessment* | *Effects* |
| --- | --- | --- |
| *Type* | RES (n = 6) | Positive (3), NS (3) |
|  | RES+END (n = 6) | Positive (2), NS (3), Negative (1) |
|  | END (n = 0) |  |
|  |  |  |
| *Supervision* | Supervision (n = 5) | Positive (2), NS (2), Negative (1) |
|  | Unsupervised (n = 3) | Positive (1), NS (2) |
|  | Mixte (n = 4) | Positive (2), NS (2) |
|  |  |  |
| *Frequency* | 1-2 sessions / week (n = 3) | Positive (2), NS (1) |
|  | +2 sessions / week (n = 8) | Positive (2), NS (5), Negative (1) |
|  |  |  |
| *Program duration* | 4-8 weeks (n = 2) | Positive (1), NS (1) |
|  | + de 8 weeks (n = 8) | Positive (3), NS (4), Negative (1) |
|  |  |  |
| *Exercise intensity* | Moderate (n = 11) | Positive (4), NS (6), Negative (1) |
|  | Individualized (n = 0) |  |
|  | HIIT (n = 0) |  |
|  |  |  |

Strength

|  | *Nombre de mesures force musculaire (MI et MS)* | *Effets* |
| --- | --- | --- |
| *Type* | RES (n = 19) | Positive (9), NS (10) |
|  | RES+END (n = 9) | Positive (5), NS (3), Negative (1) |
|  | END (n = 2) | NS (2) |
|  |  |  |
| *Supervision* | Supervision (n = 14) | Positive (9), NS (4), Negative (1) |
|  | Unsupervised (n = 5) | NS (5) |
|  | Mixte (n = 11) | Positive (5), NS (6) |
|  |  |  |
| *Frequency* | 1-2 sessions / week (n = 12) | Positive (8), NS (4) |
|  | +2 sessions / week (n = 15) | Positive (5), NS (9), Negative (1) |
|  |  |  |
| *Program duration* | 4-8 weeks (n = 8) | Positive (3), NS (5) |
|  | + de 8 weeks (n = 20) | Positive (11), NS (8), Negative (1) |
|  |  |  |
| *Exercise intensity* | Moderate (n = 22) | Positive (12), NS (9), Negative (1) |
|  | Individualized (n = 1) | Positive (1) |
|  | HIIT (n = 2) | NS (2) |
|  |  |  |

Qualité de vie

|  | *Nombre de mesures QoL* | *Effets* |
| --- | --- | --- |
| *Type* | RES (n = 9) | Positive (3), NS (5), Negative (1) |
|  | RES+END (n = 5) | Positive (2), NS (3) |
|  | END (n = 1) | Positive (1) |
|  |  |  |
| *Supervision* | Supervision (n = 6) | Positive (2), NS (4) |
|  | Unsupervised (n = 2) | Positive (1), NS (1) |
|  | Mixte (n = 7) | Positive (3), NS (3), Negative (1) |
|  |  |  |
| *Frequency* | 1-2 sessions / week (n = 6) | Positive (3), NS (3) |
|  | +2 sessions / week (n = 7) | Positive (1), NS (5), Negative (1) |
|  |  |  |
| *Program duration* | 4-8 weeks (n = 3) | Positive (1), NS (2) |
|  | + de 8 weeks (n = 10) | Positive (4), NS (5), Negative (1) |
|  |  |  |
| *Exercise intensity* | Moderate (n = 11) | Positive (3), NS (7), Negative (1) |
|  | Individualized (n = 1) | Positive (1) |
|  | HIIT (n = 1) | NS (1) |
|  |  |  |

Capacité aérobie

|  | *Nombre de mesures Aerobic and functional capacities* | *Effets* |
| --- | --- | --- |
| *Type* | RES (n = 9) | Positive (2), NS (7) |
|  | RES+END (n = 10) | Positive (4), NS (6) |
|  | END (n = 2) | Positive (2) |
|  |  |  |
| *Supervision* | Supervision (n = 11) | Positive (6), NS (5) |
|  | Unsupervised (n = 2) | Positive (1), NS (1) |
|  | Mixte (n = 7) | Positive (3), NS (3), Negative (1) |
|  |  |  |
| *Frequency* | 1-2 session(s) / week (n = 9) | Positive (4), NS (5) |
|  | +2 sessions / week (n = 11) | Positive (4), NS (7) |
|  |  |  |
| *Program duration* | 4-8 weeks (n = 7) | Positive (3), NS (4) |
|  | + de 8 weeks (n = 14) | Positive (5), NS (9), |
|  |  |  |
| *Exercise intensity* | Moderate (n = 14) | Positive (5), NS (9) |
|  | Individualized (n = 3) | Positive (1), NS (2) |
|  | HIIT (n = 2) | Positive (2) |
|  |  |  |

Nutrition

|  | *Number of assessments Oral intake* | *Effects* |
| --- | --- | --- |
| *Type* | RES (n = 3) | Positive (1), NS (2) |
|  | RES+END (n = 4) | Positive (1), NS (3) |
|  | END (n = 0) |  |
|  |  |  |
| *Supervision* | Supervision (n = 2) | Positive (2) |
|  | Unsupervised (n = 3) | NS (3) |
|  | Mixte (n = 2) | NS (2) |
|  |  |  |
| *Frequency* | 1-2 sessions / week (n = 1) | Positive (1) |
|  | +2 sessions / week (n = 6) | Positive (1), NS (5) |
|  |  |  |
| *Program duration* | 4-8 weeks (n = 3) | NS (3) |
|  | + de 8 weeks (n = 4) | Positive (2), NS (2) |
|  |  |  |
| *Exercise intensity* | Moderate (n = 6) | Positive (2), NS (4) |
|  | Individualized (n = 0) |  |
|  | HIIT (n = 0) |  |
|  |  |  |

PA level

|  | *Number of assessments PA Level* | *Effects* |
| --- | --- | --- |
| *Type* | RES (n = 4) | Positive (3), NS (1) |
|  | RES+END (n = 5) | Positive (2), NS (3) |
|  | END (n = 0) |  |
|  |  |  |
| *Supervision* | Supervision (n = 0) |  |
|  | Unsupervised (n = 5) | Positive (1), NS (4) |
|  | Mixte (n = 4) | Positive (3), NS (1) |
|  |  |  |
| *Frequency* | 1-2 sessions / week (n = 3) | Positive (1), NS (2) |
|  | +2 sessions / week (n = 6) | Positive (3), NS (3) |
|  |  |  |
| *Program duration* | 4-8 weeks (n = 4) | Positive (2), NS (2) |
|  | + de 8 weeks (n = 5) | Positive (2), NS (3) |
|  |  |  |
| *Exercise intensity* | Moderate (n = 7) | Positive (4), NS (3) |
|  | Individualized (n = 1) | NS (1) |
|  | HIIT (n = 0) |  |
|  |  |  |

Outcomes / patient characteristics :

Muscle Mass

|  | *Nombre de mesures masse maigre / musculaire* | *Effets* |
| --- | --- | --- |
| *Type de tumeur* | Digestive (n = 4) | Positive (2), NS (2) |
|  | Head and neck (n = 4) | Positive (2), NS (2) |
|  | Various (n = 3) | Positive (1), NS (2) |
|  |  |  |
| *Age* | Older than 63 years (n = 4) | Positive (1), NS (3) |
|  | Younger than 63 years (n = 7) | Positive (4), NS (3) |
|  |  |  |
| *Sex* | More than 36% of women (n = 5) | Positive (3), NS (2) |
|  | Less than 36% of women (n = 7) | Positive (2), NS (4), Negative (1) |
|  |  |  |

Strength

|  | *Nombre de mesures masse maigre / musculaire* | *Effets* |
| --- | --- | --- |
| *Type de tumeur* | Digestive (n = 15) | Positive (9), NS (5), Negative (1) |
|  | Head and neck (n = 4) | Positive (2), NS (2) |
|  | Various (n = 11) | Positive (3), NS (8) |
|  |  |  |
| *Age* | Older than 63 years (n = 10) | Positive (4), NS (5), Negative (1) |
|  | Younger than 63 years (n = 20) | Positive (10), NS (10) |
|  |  |  |
| *Sex* | More than 36% of women (n = 14) | Positive (9), NS (5) |
|  | Less than 36% of women (n = 16) | Positive (5), NS (10), Negative (1) |
|  |  |  |

Quality of Life

|  | *Nombre de mesures masse maigre / musculaire* | *Effets* |
| --- | --- | --- |
| *Type de tumeur* | Digestive (n = 6) | Positive (2), NS (3), Negative (1) |
|  | Head and neck (n = 3) | Positive (1), NS (2) |
|  | Various (n = 6) | Positive (3), NS (3) |
|  |  |  |
| *Age* | Older than 63 years (n = 5) | Positive (2), NS (3) |
|  | Younger than 63 years (n = 10) | Positive (4), NS (5), Negative (1) |
|  |  |  |
| *Sex* | More than 36% of women (n = 8) | Positive (4), NS (4) |
|  | Less than 36% of women (n = 7) | Positive (2), NS (4), Negative (1) |
|  |  |  |

Aerobie capacity

|  | *Nombre de mesures aerobic and functional capacities* | *Effets* |
| --- | --- | --- |
| *Type de tumeur* | Digestive (n = 9) | Positive (5), NS (4) |
|  | Head and neck (n = 3) | Positive (1), NS (2) |
|  | Various (n = 9) | Positive (4), NS (5) |
|  |  |  |
| *Age* | Older than 63 years (n = 9) | Positive (4), NS (5) |
|  | Younger than 63 years (n = 12) | Positive (4), NS (8) |
|  |  |  |
| *Sex* | More than 36% of women (n = 8) | Positive (3), NS (5) |
|  | Less than 36% of women (n = 13) | Positive (5), NS (8) |
|  |  |  |

Nutrition

|  | *Nombre de mesures oral intake* | *Effets* |
| --- | --- | --- |
| *Type de tumeur* | Digestive (n = 2) | Positive (2) |
|  | Head and neck (n = 1) | NS (1) |
|  | Various (n = 4) | NS (4) |
|  |  |  |
| *Age* | Older than 63 years (n = 3) | Positive (1), NS (2) |
|  | Younger than 63 years (n = 4) | Positive (1), NS (3) |
|  |  |  |
| *Sex* | More than 36% of women (n = 3) | NS (3) |
|  | Less than 36% of women (n = 4) | Positive (2), NS (2) |
|  |  |  |

PA level

|  | *Nombre de mesures PA level* | *Effets* |
| --- | --- | --- |
| *Type de tumeur* | Digestive (n = 3) | Positive (1), NS (2) |
|  | Head and neck (n = 1) | Positive (1) |
|  | Various (n = 5) | Positive (2), NS (3) |
|  |  |  |
| *Age* | Older than 63 years (n = 4) | Positive (1), NS (3) |
|  | Younger than 63 years (n = 5) | Positive (3), NS (2) |
|  |  |  |
| *Sex* | More than 36% of women (n = 6) | Positive (2), NS (4) |
|  | Less than 36% of women (n = 3) | Positive (2), NS (1) |
|  |  |  |
